# Psychological Impact and Influence of Animation on Viewer's Visual Attention and Cognition: A Systematic Literature Review, Open Challenges, and Future Research Directions

**DOI:** 10.1155/2022/8802542

**Published:** 2022-08-31

**Authors:** C. K. Praveen, Kathiravan Srinivasan

**Affiliations:** ^1^VIT School of Design, Vellore Institute of Technology, Vellore 632 014, India; ^2^School of Computer Science and Engineering, Vellore Institute of Technology, Vellore 632 014, India

## Abstract

Animation is an excellent method to associate with the audience in a fun and innovative manner. In recent span, animation has been employed in various fields to enhance knowledge, marketing, advertisement, and age groups from infants to adults. The present communication expounds the systematic review on the impact created by animation on the viewer's visual attention. For this review, a database such as Google Scholar, ScienceDirect, Taylor & Francis, and IEEE Xplore were pursued for publications on the impact of animation on viewer's visual attention from January 2015 to December 2021. The search results showcased 175 titles with 114 full articles, out of which 35 were related to viewers' visual attention towards animation. These reviewed studies comprised of physical outcome (*n* = 9), psychological outcome (*n* = 15), and cognitive outcome (*n* = 11) from which the attention-related factors, physical effects, and cognitive effects of animation were assessed. The animation has influenced the viewer's visual attention through the integration of the different stimuli and the highly organized presentation. Furthermore, the animation has also aided the viewer in attaining greater conceptual understanding, thereby facilitating their cognitive response. As a result, the animation was found to be helpful in enhancing learning skills, food marketing, and teaching strategy. Furthermore, the drawbacks and future recommendations of the studies were elaborated. In addition, challenges and open issues faced during the studies were discussed. Finally, the priority areas in animation identified for promising future directions to visualize large pool data, provide smart communication, and design 3D modeling structures were highlighted.

## 1. Introduction

Animation is a comprehensive introduction to animated films, from cartoons to computer animation. In layman's terms, it can be described as a state of being full of life. It brings the life of unanimated objects to moving objects, thereby attracting the modern world with its features [1]. In other words, it is a form of pictorial presentation that has become the most prominent feature of technology-based learning environments. In the modern world, it has become an essential tool for presenting multimedia materials for learners to understand them better [2].

**Figure 1:**
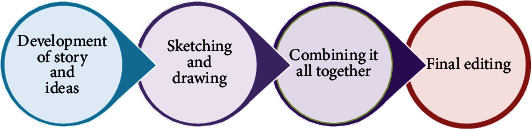
Process of animation.

Animation techniques have been developed over a while, either in 2D drawings or 3D objects like clay, stop motion, or motion graphics. It has become a reliable and significant platform for various fields that have impacted viewers' visual attention through its magic. The animation need not be a full-length movie to attract the viewers' visual attention; rather, it can be a clip of a few seconds comprised of just a few frames. The animation videos can be processed as represented in Figure 1.

An idea of integrating traditional animation with the digital 2D animation technique was proposed by Purwaningsih [3]. It provides an alternative pipeline for hand-drawn 2D animation shorts, thereby optimizing the production time.

The impact of animation on viewer's visual attention and attention span was reviewed and reported either with respect to animated characters or character motion. The research on considering both as influencing factors for viewer's visual attention is insignificant. The present survey focuses on how animated characters and their motions create an impact on the viewer's visual attention. Also, it emphasizes the physiological and cognitive impact created by the animation on the viewers.

### 1.1. Need and Objectives of the Study

Animation is capable of attracting a large audience in every field. As a result, most people are exposed to this interesting field of knowledge. The major objectives of this systematic review are summarized as follows:

- To exemplify the impact and influence of animation and animated characters on viewers visual attention and cognition
- To elucidate the various approaches and techniques used in attention based animation studies
- To elicit the standards, regulations, guidelines, and best practices that could assist the animation professionals in understanding the viewers' cognitive behavior
- To exemplify the current trends and open issues of the impact of animation on viewers' attention and cognition
- To elucidate the future research directions in animation-based attention studies

### 1.2. Related Work

Etemad et al. [4] have analyzed the motivating factors for processing motion features and their relative degrees of significance in a general paradigm called the perceptual validity (PV) model. The PV consists of four components: association, contextual dependency, internal consistency, and external consistency with underlying elements (bodily action, bodily expressions, facial action, and facial expression). A case study was conducted with this paradigm based on the contextual dependency and finally discussed with Disney's principles of animation. Zong et al. [5] have discussed the importance of character expression shaping in animated films. The features of facial expression design, such as exaggeration, accuracy, and virtuality, were briefed. Likewise, the expression techniques such as association, personification, exaggeration, and deformation were discussed. Finally, a case analysis of animation expression shaping with respect to every character depicted in the Kung Fu Panda film was carried out in-depth.

Kim et al. [6] studied character-audience similarity's impact on evaluating public service announcements (PSAs). The characters of smokers and persuaders are differentiated to explore their different roles in message effectiveness. Shao [7] has discussed the performance of visual humor in animation from the point of view of image, color, action, and rhythm. The image of an animated character is suggested as a bearer of visual humor. It suggested that the humor can be enhanced/created either in the form of body proportion (genius rat in Ratatouille) or structural reorganization (Pigsy's head in Journey to the West). The animated character's color is considered to render emotional visual humor (Panda Po in Kung Fu Panda). The action of the animated character is proposed to be surreal humor (Tom cat and Jerry mouse in Tom and Jerry). Finally, the rhythm of animated films was proposed to affect the audience's visual and psychological feelings.

Shah et al. [8] studied the application of animation in pharmaceutical advertisements and its impact on consumer perception of the risks and benefits of the drug. Two sets of studies have been carried out for the analysis. Rotoscoping was used to test the effects of animation in this study. Study 1 was carried out to assess whether any shift in perception exists and whether it agrees with a memory effect. In study 2, the findings from study 1 were extended by including consumer implications in order to demonstrate the downstream consequences of the use of animation in pharmaceutical advertisements. Smith and Neff [9] have investigated the influence of animated gestures in controlling personality perception. A sequence of four diverse gestures with twelve motion adjustments was selected as stimuli for the study. The correlation in personality perception was determined. In addition, the potential and possible limits of motion editing approaches were discussed. Two constellations of motion adjustments were selected for the study.

Vijayakrishnan et al. [10] analyzed the importance of animated cartoon characters in product marketing through advertisement. The preference of children over products having cartoon characters was scrutinized. The strategies used in the global market for selling the products using animated characters were also discussed. Geal [11] has explored how animation can manipulate a reflexive intertextual framework related to religious prohibitions on artistic mimesis that might replicate and threaten God's creative act. The limitations of the existing survey are listed briefly in Table 1.

**Figure 2:**
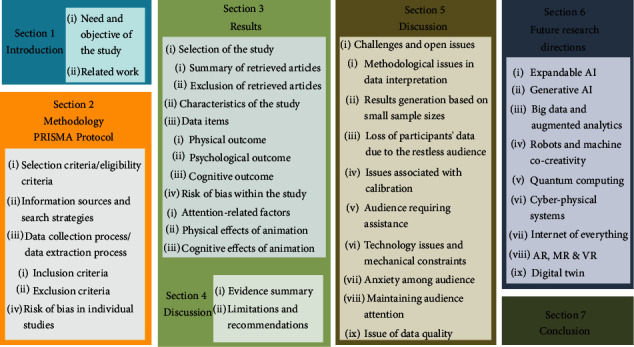
Structure of this review.

This paper is divided into seven sections and its general layout is depicted in Figure 2. The first section introduces the animation, its impact on the viewer's psychology, and attention span. It also briefs the objective of this study, the limitations of the existing research, and the present study's contribution. The second section explains the application of the PRISMA protocol to evaluate the reviewing of other types of research. The evaluation is based on the survey's selection criteria, its information sources and search strategies, data collection process, and risk of bias in individual studies. The third section elaborates on the selection of the present study, its characteristics, selected data items, and the risk of bias within the studies. It also assesses animation's attention-related factors and physical and cognitive effects. The fourth section briefly presents the summary along with the limitations and recommendations. The fifth section elaborates on the challenges and open issues the researchers face during the study. The sixth section highlights the future research directions in the field of animation. Finally, the last section summarizes all the facts and concludes the reviewed results.

## 2. Methodology

### 2.1. PRISMA Protocol

The present study is reviewed based on the PRISMA (Preferred Reporting Items for Systematic Reviews and Meta-Analyses) Protocol [22, 23]. It is a set of recommendations designed for reporting systematic reviews. These guidelines aid authors in improving the reporting of systematic reviews and meta-analyses and ensuring the accuracy and transparency of the studies reported [24]. The present study's reporting quality can be optimized by completing the review report based on the PRISMA–P statement and checklist. Moreover, it also improves the efficiency of the peer review process and enables the readers to get a clear view of the author's work.

**Figure 3:**
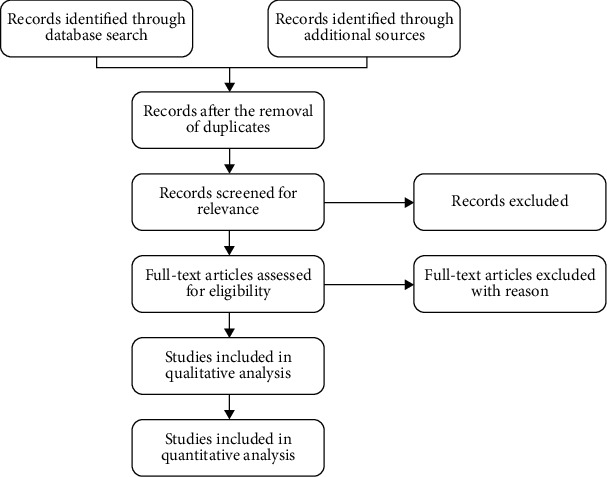
Flow diagram of PRISMA protocol.

The steps followed in the PRISMA protocol are represented in Figure 3. It is provided briefly as follows: (a) Identification, the records are identified through database searching and additional sources. (b) Removal of duplicates, the records that appear more than once should be removed to avoid reviewing the duplicate records. The entire list of records is exported to a citation manager to remove the duplicate records. The remaining records are entered in the second top box. (c) Screening, the number of screened articles are entered in the following box. Furthermore, this value will be the same as that of the number entered in the duplicate removed box. Further, the articles are screened based on their titles and abstracts. The number of articles excluded in this screening process is recorded in the relevant box. (d) Eligibility, the number of excluded articles after the screening process is subtracted from the total number of records screened. Full-text articles are assessed for eligibility. All these full-text articles are eligible for the final reviewing process. The number of excluded articles at this point is recorded in the appropriate box. (e) Inclusion, the number of excluded articles is subtracted from the total number of articles reviewed for eligibility. Furthermore, this number is entered in the qualitative analysis box. The number of studies list is entered in the quantitative synthesis box to perform the meta-analysis.

#### 2.1.1. Selection Criteria/Eligibility Criteria

The criteria selected were defined before undergoing screening of any articles. The selection criteria are listed in Table 2.

The criteria selection helps to limit the broad topic to direct relevance for the research questions. The language is selected as English as it is the primary publication language for scientific articles. The year of publication is limited to providing a review based on recently published research works. Finally, peer-reviewed articles are considered to provide good quality of work and confirmed results. Also, published thesis work is considered, providing more detail about the research work introduced in peer-reviewed articles by the same or similar authors. Animation-based attention-creating articles were selected for the reviewing process.

#### 2.1.2. Information Sources and Search Strategies

The search databases selected for article retrieval should have good coverage of the body of the relevant work. For this purpose, the two major exiting multidisciplinary databases, Web of Science and Scopus, were selected. Also, scientific databases like Google Scholar and ResearchGate are included as they cover good reporting of animation-related attention-creating articles. In recent times, these research articles can also be retrieved from general databases. However, Google Scholar gains superiority due to its positive correlation with the citation counts from various sources. Many of the works relevant to animation-based attention-creating articles can be retrieved from this database. The publishers such as IEEE, ScienceDirect, Springer, and SAGE also provide direct access to their publications, and their databases were also assessed for their yield of additional relevant results. All the relevant papers can be expected to be available online as the year of publication selection is from 2015 and above. So the analogue search was not conducted separately. Therefore, the electronic database searches were executed from January 2015 to the year 2021 until the preparation of the review. The reference list of all the relevant articles was analyzed for their significance with the research objectives and screened accordingly. The same selection criteria were applied here.

The search strategies need to be fine-tuned to get a better search of articles. Meanwhile, it should expose all relevant research works under a manageable level with no increase in the overall workload of the reviewing process. For the given research objective, the attention-creating articles published in the field of animation were chosen. The research terms for the search were used in either form of individual keywords or a combination of keywords. And specifically, the research terms used were ‘animation' OR ‘impact of animation' ‘Animation' AND ‘psychology' OR ‘animation' AND ‘audience' OR ‘animation' AND ‘cognitive psychology' OR ‘animation' AND ‘audience visual attention'.

#### 2.1.3. Data Collection Process/Data Extraction Process

The title of the articles retrieved from the databases is evaluated for their significant relevance to the research objectives. Furthermore, their respective abstracts are read thoroughly. Based on this, the most relevant articles were segregated and organized in a Microsoft Excel sheet.

*(1) Inclusion Criteria*. Inclusion criteria for this study include the year of publication, country of origin, methodological base, experimental context, sample characteristics, study duration of existing articles, outcome measures, and exposure to animation duration.

*(2) Exclusion Criteria*. The criteria excluded for this study comprise lack of access to the full article, unsuitable research articles, letters to the editor, and retraction articles review articles.

The study's key findings mainly focused on how effective the animation in the existing articles. And no attempts are made to contact the authors for missing details in their respective articles.

#### 2.1.4. Risk of Bias in Individual Studies

All the articles were independently evaluated based on the inclusion and exclusion criteria to assess the risk of bias in individual studies. The information extracted from each study is evaluated using the quality assessment tool. For the effective quality assessment, the checklist is made based on the following criteria: yes, no, not applicable (NA), and not reported (NR). The checklist of quality assessment tools includes the following criteria mentioned in Table 3.

## 3. Results

### 3.1. Selection of the Study

#### 3.1.1. Summary of Retrieved Articles

The summary of the search databases visited and the number of articles obtained from the respective sources is presented in Table 4. Further, this table shows the percentage of articles retrieved from each academic database and reveals that the highest number of articles were retrieved from Google Scholar (*n* = 199). It comprises research articles, conference papers, and students' dissertations. Other databases like Springer, Science Direct, and Taylor and Francis account for 6.76%, 6.41%, and 6.05% of the total number of articles. The rest of the articles were retrieved from IEEE Xplore (4.27%), ResearchGate (3.20%), and Wiley Online Library (2.49%).

#### 3.1.2. Exclusion of Retrieved Articles

The number of articles retrieved from the search database was reduced with the following eliminated procedure based on PRISMA protocol.

(i) Elimination of the articles based on language (*n* = 6), irrelevant titles (*n* = 75), and reduction of duplication (*n* = 25) from various search databases, leading to reduction from 281 to 175
(ii) Elimination of articles after the examination of abstracts, leading to the reduction of articles from 175 to 114
(iii) Elimination of articles based on thoroughly reading the full article led to a reduction from 114 to 35. The articles were eliminated for the following reasons:
  - Report on animation impact, 37
  - Not focused on animation, 18
  - Report on animation application, 14
  - Case study, review, and others, 5
  - Not enough information, 5

**Figure 4:**
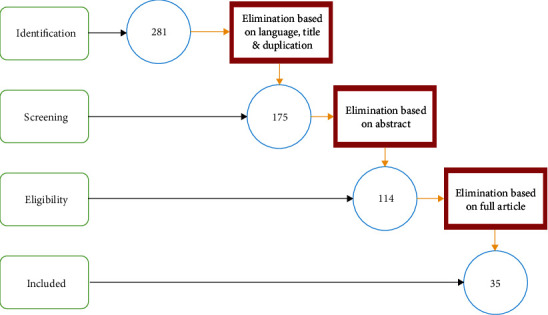
Elimination process of articles based on PRISMA protocol.

The procedure flow for selecting articles for the study is depicted in Figure 4, which shows the elimination procedure.

### 3.2. Characteristics of the Study

Based on the selection process, 35 articles were shortlisted for the systematic review. Each article was reviewed, and the information gathered from it was tabulated. The following information was extracted from the articles: description of the study, sample and design applied in the study, type and duration of animation used in the study, and outcome and findings of the study and case-control applied within the study. The study's characteristics, as itemized above, are summarized in Table 5.

### 3.3. Data Items

The study participants' ages ranged from 30 months to 30 years, and most of the studies included both sex samples, with the exception of three studies with female samples alone and gender not mentioned in eight studies. In addition, most of the studies included 3D animation (*n* = 10) followed by 2D animation (*n* = 6) and flash (*n* = 4), and the remaining studies included motion graphics, VR, and AR. Further, the outcomes reviewed from all these studies, namely, physical outcome (*n* = 9), psychological outcome (*n* = 15), and cognitive outcome (*n* = 11), are presented in Table 5.

#### 3.3.1. Physical Outcome

The importance and necessity of physical exercise was easily delivered to the primary grade students. The results showed a significant difference in self-efficacy, learning, benefits, importance, personal best, and fun between the control and experimental groups (*p* ≤ 0.05) [31]. The hand manipulative tasks was made better with the help of animation. The results showed that the animation groups ranked their difficulty levels (cognitive load) significantly lower than the static groups. Moreover, viewing hand or not made no difference for the animation group [36].

The effects of visual cueing depend on the subject matter and the learner's learning strategies [40]. The pretest scores revealed insignificant scores between the test and control groups. However, there is a significant difference between the test and control group in the sequential memory test [55]. The microintervention study revealed the positive impact of animation on creating awareness on body image among the adolescents. It helped them to understand the importance of telling a bully to stop. The study results showed a significant difference in body satisfaction between the groups. However, it is insignificant to media literacy and self-efficacy [29]. There is a significant difference in the learning outcomes between each PK (prior knowledge) group for reading comprehension. The animation annotation was easily noticed by the low PK group, whereas the text zone was noticed by the high PK group [38]. The results obtained from SPQ and BMI revealed the following results: Pororo - So-Yang type boy, Petty - So-Yang type girl, Loopy - So-Eum type girl, Pobby & Harry -most obvious contrast [50]. There is a significant difference between lip-syncing and gaze to target for perceived speech intelligibility [43].

#### 3.3.2. Psychological Outcome

Food marketing industries have efficiently utilizing animation as a tool to attract the children, and they were assessing their attention towards healthy/unhealthy food items. Children are attracted to the food and beverages product with or without animated characters. Children were significantly chosen the less healthy product with or without character. Children significantly preferred more or less healthier products irrespective of character [51]. Children's pupil diameter increased on watching the candy condition. However, no significant difference was observed in the children's visual attention or emotional arousal towards candy or food conditions. There is a significant difference in children's emotional arousal to unhealthy products due to the parent's restriction of candy at home [32].

The children recalled the story and more content words significantly from animated conditions than a static conditions. Children's visual attention was significant with animated conditions compared to static conditions [57]. The children were able to recognize the facial identity through dynamic facial animation. However, they failed to learn the facial expression. There is no significant difference observed between the pre- and post-familiarization tests [47].

The animation has delivered a better opportunity to have self-awareness and knowledge on the health issues without any hesitation. The implementation of computer-animated agent provides assistance to deliver personally relevant information on breast cancer. It helps to reduce anxiety, support psychological needs, and boost confidence. The results showed a significant difference in the proportion of participants with unanswered questions for the post-intervention period [30]. The health awareness regarding the conditions of glaucoma was perceived by the patients through animation video. There is a significant difference in the patients' knowledge scores between pre- and post-intervention (*p* ≤ 0.001). Rural residence, low income, and unemployment were identified as influencing factors for acquiring glaucoma knowledge [45].

There is a positive correlation between the learning experience between the VR simulation and traditional practice [52]. There is no significant difference between the real and hybrid CG characters (*p* = 1.00). A less significant difference existed between real and CG characters (*p* < .001) as well as between CG and hybrid characters (*p* < .001). The CGI could feature the actor those who are alive or dead and are capable of enhancing the parasocial interaction and relatability [25]. The animated character influenced the viewer's attention. There is a significant difference in eeriness between the Pixar character and the Toon character (*p* < 0.05). There is a significant difference in eeriness between the photorealistic human character and the Toon character (*p* < 0.05) [48].

The prior knowledge about the techniques behind the making of stop motion films may influence the impact of viewer's attention towards the technical aspects rather than focusing on the story. However, the viewer's attention can be engrossed in the film, and it may develop a deeper connection with the story [26]. The level of exaggeration in animated cat is insignificant to the audience's perception of the appeal of the realistic feline character. Moreover, the significance of believability is higher for high exaggeration clips than for low exaggeration clips [37]. The frequency of exposure to animated television cartoons is higher among females, and it is greatly influenced based on their level of education. The perception of such cartoons varies with the level of education [56]. The viewer's pleasantness feeling toward animated character design aesthetics is insignificant to their gender and age group [44].

The animated virtual ads attracted the participants than the static ads. The virtual ads presented in the nonbattle scene attracted the participants than those ads in the battle scene. The interaction effect between ad animation and in-game context on fixation count is insignificant [41]. The animation intensity and animation color on the sponsorship signage showed negative effect on the viewer's attention. The arousal of the viewer's confusion due to increased levels of animation intensity was explored. And the results showed an insignificant effect of animation intensity on viewer confusion [46]. Also, there is no significant difference in color [59].

#### 3.3.3. Cognitive Outcome

The studies reported that animation played an essential role in the cognitive development of children (*n* = 6). The children who read the AR storybook were more confident in retelling and recalling the story when compared to those who read its printed version [27]. The mother's video prompted larger pupil dilations and a more smiling and cheerful eye blinking rate among the infants. The highest value cartoons prompted long looking time, reduced blinking, but no increased smiling or pupil dilation [34, 40]. Animated films positively affect a child's involvement in symbolic mediation and the level of arbitrary behavior [54]. Also, it was observed that the executive functions of the preschoolers were disrupted after watching the animated fantastical events [28]. A significant effect of animated features in ebooks (motion and sound) was observed on children's vocabulary acquisition, story retelling, and visual attention [35]. There is no significant difference in birth weight, age, parental educational level, or preintervention performance levels between the groups. The trained group showed tremendous results immediately after the training and at 6 weeks follow-up [42].

Some of the studies reported the role of animation in the teaching field (*n* = 5). The adoption of 3D animation as a teaching tool for illustrating surgical skills in medical education was investigated. The test scores showed higher significance for the 3D animation group when compared to the traditional teaching group (*p* < 0.0001) [33]. The animation lecture with instructional design helps in guiding learner's attention, thereby making them focus on the important instructions in the instructions. The animated group required less cognitive load, and they outperformed on the open-ended questions. It was further confirmed with insignificant differences between the two groups in the Genetic Foundation Test [39]. Idioms learning can be made easier by watching an English animated movies. There is a significant effect on learning idioms through English animated movies (*p* < 0.05) [49]. The spatial features of the animations and the simulations facilitated the development of spatial ability of the 12th grade students. The experimental group's spatial ability and reasoning skills have higher significance than the control group (*p* < 0.05) [53]. The cueing by pedagogical agents positively affected learning performance and instructional efficiency. The cognitive load measures between the two groups were insignificant [58].

### 3.4. Risk of Bias within the Study

The risk of bias assessment within the studies is summarized in Table 6. The criteria for the assessment were based on the study design and data analysis. Nearly all the study participants were randomly selected (*n* = 28) with control group (*n* = 15) assigned. Some of the studies were conducted in isolation (*n* = 31), and the pretest and post-test (*n* = 16) method was employed to assess the significance of the hypothesis developed. The participants' visual perception of the animation was determined by their capability to recall or retell (*n* = 27). The data obtained in most of the studies were analyzed using power analysis (*n* = 12), validity measures (*n* = 10), and baseline comparisons (*n* = 7) and were employed in some studies. Follow-up on the influence of the animation was further assessed in a few studies (*n* = 3), and missing data were reported in a few studies (*n* = 5).

### 3.5. Attention-Related Factors

Some of the studies reported in this review are solely concentrated on the visual attention of the participant's towards animation (*n* = 12). The attention-related factors among these studies are animation's interactive features (*n* = 3), intensity (*n* = 1), design (*n* = 1), motion (*n* = 3), sound (*n* = 1), annotation (*n* = 1), and character (*n* = 3). The factors that are insignificant with the viewer's visual attention was animation's color.

In twelve out of thirty-five papers, eye-tracking technology was employed to assess the participants' visual attention to the animation. The pupil movement and fixation time was observed to assess the viewer's attention towards the animation.

### 3.6. Physical Effects of Animation

Animation has created awareness among adolescents about their body images and provided knowledge about the necessity of physical activity. It also helped women get a detailed report on mammographic procedures without hesitation. Moreover, it also delivered a knowledge on the health issues related to glaucoma.

### 3.7. Cognitive Effects of Animation

As mentioned earlier, the animation has created some cognitive effects towards infants to adults. The animated ebook has helped the children understand the story's structure and content. Furthermore, animation made it easy to learn the surgical procedures like intercostal drain insertion and suprapubic catheter insertion. Also, the concepts of genetics, such as cell division, mitosis, and meiosis, were presented in animation, and the participant's performance was found to be improved. Moreover, the student's spatial ability and reasoning skills were improved by watching the animation lectures.

## 4. Discussion

### 4.1. Evidence Summary

From the overall studies, it was evident that the animation was employed in various applications to attract and assess the viewer's attention. Among thirty-five studies, five briefed about the animated characters and one study about the animation motion.

The rest of the studies described the perception of audience towards implementing animation in the following phenomenon: learning skill improvement (*n* = 15), teaching strategy (*n* = 2), health awareness (*n* = 5), advertisement (*n* = 3), food marketing (*n* = 2), validating hearing aid (*n* = 1), and political awareness (*n* = 1).

### 4.2. Limitations and Recommendations

Although the studies reported in this survey showed a significant difference and the hypothesis generated was accepted, some limitations still exist. The common limitations identified in the studies are short period of time for implementation [53], smaller sample size [27, 37], nongeneralizability [25, 27, 32, 39, 53], nonrandomization trials [52], and no control group and post only group [45]. Few other studies have reported the possibility of cross-contamination among the control and experimental group [33], increased dropout of participants before completing the post-test questionnaire [52], and underestimation of participant's knowledge of expressing words which might directly affect the animation [57].

Arshad et al. [44] have examined the “Pleasure” as a sole emotional response to describe the pleasure level of human emotion towards the Malaysian animation cartoon characters. In contrast, the PAD (pleasure, arousal, and dominance) model utilized in the study has two other dimensions: arousal and dominance.

The audience could not feel the warmth of the real human character in the animated short film as the animation span is too short [48]. In another study, there is a possible way for the audience to have different perceptions regarding the meaning of the word “believability.” Moreover, the cat's exaggerated motion alone studied might express the intrinsic characteristics of its particular character design [37]. While studying the viewer/character relationships, the PSI (parasocial interaction) scores remained low, which may be due to the cause that it features nonhuman characters in all-CGI conditions. At the same time, the other conditions featured only humans [25].

Some of the typical future recommendations mentioned in the studies are an extension of the study period [53], increasing the study sample size [37] and implementing a randomized sample approach from various situations to overcome the limitation of result generalization [37, 52, 53].

The audience's perception of various anthropomorphic animal characters performing various actions in different situations should be examined [37]. In addition to the animation, the story's narration is more concentrated when designing a storybook app [57]. Moreover, TV animated cartoons can be designed to attract people with tertiary education for political promotions and political mobilization [56].

## 5. Challenges and Open Issues

**Figure 5:**
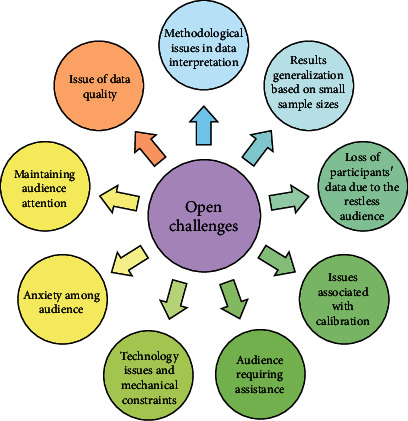
Challenges and open issues.

The challenges and open issues faced by the researchers during the study are elaborated in this section, and it is shown in Figure 5.

### 5.1. Methodological Issues in Data Interpretation

The methodological issues in data interpretation may occur due to animation completion time, fixation duration, and other confounding variables. Fixation duration may be employed to determine the participant's eye or head movements. Mostly eye-tracking devices and gaze movement trackers are utilized for this purpose. Any fault with these devices will affect the data quality, data loss, and data interpretation bias. Li et al. [28] have suggested that fixation data points showed the preschooler's more significant mobilization and limited processing capacity. Tummeltshammer et al. [34] have determined the unfiltered eye movement data using SMI's BeGaze analysis software to overcome the error caused by the tracking device or participants in attention.

### 5.2. Results Generalization Based on Small Sample Sizes

The generalization of results based on small sample sizes may not be appropriate for all the cases. Most of the studies mentioned it as a limitation due to various concerns such as participants' demographical features and socio-economical features. Al-Balushi et al. [53] have reported improving logical thinking and spatial thinking skills of 12th grade students of Oman. He has also stated that further investigations are required due to the small sample size. Danaei et al. [27] have reported that the children who read the AR storybook were more confident in retelling and recalling the story.

The specific format or instructions employed in the research can also affect the generalization among the same or different populations. For instance, a specific pedagogical agent format that shows attraction towards the younger population might not show the same effects on adolescents and adults [58]. Similarly, the instructions designed to visualize in animation may not be appropriate for visualizing the same in real phenomena [39]. Binder et al. [32] suggested conducting more eye-tracking experiments with integrated food cues to attract children's attention toward healthy foods.

### 5.3. Loss of Participants' Data due to the Restless Audience

The audience becomes restless when the study duration is too long. This may be overcome by regular contact with them or follow-up studies. In some cases, participants find it difficult to spare their free time voluntarily. For instance, many students find it difficult to complete the questionnaire in their free time due to the stressful semester [52]. Sometimes, it is difficult to compel the participants to make things if they are children or infants. Among 39 children, one refused to retell the comprehensive stories learned through animated storybooks, so the corresponding data was removed from the study [57]. Two children in an experimental group refused to participate during the middle of the study [55].

In some cases, the data will be collected indirectly through random websites or by issuing pamphlets to random people. For example, Okoro and Onakpa [56] have collected data from selected towns in North Central Nigeria by issuing 385 copies of the questionnaire. Among these, 2% of the questionnaire were not received due to the restless audience.

### 5.4. Issues Associated with Calibration

The calibration of devices employed for measuring participants' visual attention plays a vital role in acquiring good quality data and aids in providing a better focus on the participants. Unfortunately, due to poor calibration (> 1) of the eye-tracking device, deviated results were obtained from five children, which may negatively impact the overall results [32].

### 5.5. Audience Requiring Assistance

The most important challenge faced in the study is to find the audience who requires assistance to participate in the study. Furthermore, this might help in acquiring better and more appropriate results. For example, Danaei et al. [27] have identified and helped the children who had struggled to start retelling the story learned through AR story book. And they were encouraged to continue the story.

### 5.6. Technology Issues and Mechanical Constraints

The technological issues may be caused due to unavoidable faults in the devices employed in the study. Due to this issue, the data points measured will be low, resulting in removing those data points. For example, in a study by Hendrikse et al. [43], the electrodes reached saturation due to the loose connection in the EOG (electrooculogram) electrodes for some participants. This, in turn, affected the data quality, and the corresponding data point was removed until it was adjusted to drift compensation. Similarly, two children were excluded from the study due to the problem that occurred in the stimulus presentation of fruit and candy [32].

### 5.7. Anxiety among Audience

Anxiety among the audience is another challenge faced during experimenting with new ideas. For example, al-Balushi et al. [53] have attempted to teach the critical concepts of 12th grade chemistry through animation. However, the students facing stressful periods due to the important exam in 12th grade made them anxious about the adoption of new teaching technology, which negatively affected the results. Nevertheless, the study still showed healthier results with improved spatial ability and reasoning skills of those students.

### 5.8. Maintaining Audience Attention

Another challenge in making animation successful is maintaining the viewer's attention. Attention can be influenced by various factors like animation span, animation intensity, animated character, the motion of the animated character, and sound. Likewise, it may be affected by intervening factors such as restlessness of the audience in the real-time study settings and eye irritation. This situation can be overcome by conducting studies in a silent room where the audience can focus on the visual animation without getting distracted by external factors [49]. In some cases, the audience may get distracted by the instructions provided in the animation video [39].

### 5.9. Issue of Data Quality

One of the significant issues faced while carrying out studies is the quality of data obtained. And it may be influenced by the missing data due to an error in the instruments employed. It can either affect the result or may be corrected. For instance, due to the eye-tracking device's problem, children's eye movements were not clearly captured, which resulted in extremely low fixation time [57]. Similarly, 31.4% of EOG data were missing due to some error in the device. However, the missing data points are adjusted by entering them as not-a-number in the analysis [43]. Figure 5 illustrates the open challenges associated with this research.

## 6. Future Research Directions

**Figure 6:**
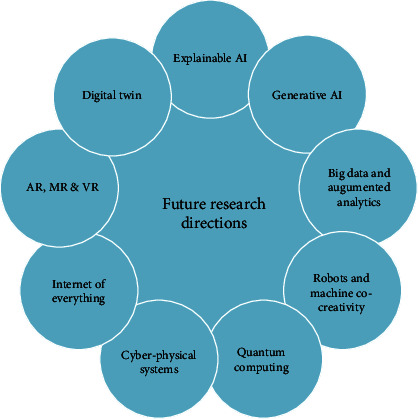
Future research directions.

The priority areas identified for future research directions are elaborated to strengthen the body of evidence. These include the advanced applications of animations that may make life easier and are listed in Figure 6 and are elaborated below.

The foremost application could include artificial intelligence (AI) that may generate 3D motion from video without any capture equipment [60]. The AI and advanced hardware can bring breathing life into animation by blurring the lines between the virtual and real characters. The application of AI into animation has reduced the post-production time, limited the need for character design, and aids in improved lip-syncing [61]. The explainable AI is an artificial intelligence operation that runs on deep neural networks. The practical applicability and promotion of the AI tool are enhanced by developing computational help [62]. The major challenges in AI are to succeed explainability in its program, which can be facilitated with animation techniques [63]. The explainable AI can be adopted in autonomous car decision-making and energy efficiency in smart homes [64] and medical imaging [65, 66]. Meanwhile, generative AI is a machine learning algorithm that can generate new content through text, images, and audio content. In addition, it can generate human-like language output [67].

Analyzing a large amount of fragmented data can be simplified by converging the big data and augmented analytics. Moreover, it helps to provide simplified statements to the customers in an understandable manner [68]. The visualization of a large pool of data can be made easier with the help of animation. Moreover, the data visualization can be integrated with augmented and virtual reality [69]. The big data and augmented analytics play a major role in video gaming. For instance, Pokemon Go is a location-based Japanese video game franchise. This game transforms the gamer's physical location into an augmented world where the characters are superimposed on the reality seen through their mobiles. The Global Positioning System (GPS), a major staple of big data, makes this possible by allowing data collection and storing it upon the crowd-sourced data [70].

Quantum computing deals with pulling together the theoretical ideas of computer science and fundamental physics. It has been the focus of many large companies such as Google, IBM, and Microsoft. The algorithm created from quantum computing concepts can be employed to design a 3D modeling [71]. It is based on the qubits that give rise to new logic gates, which enable constructing a new algorithm. However, it is still in its emerging phase, and for future development, it is necessary to overcome the obstacles like decoherence and scalability issues [72].

The collaboration of robots and machines to perform a day-to-day task will be the perception of the modern era. However, its ethical issues are yet to be analyzed and eradicated [73]. Internet of Everything (IoE) provides interconnection of physical items to frame an information network that provides smart communication services to the users. The IoE finds applications in the fields like health care, smart grids, smart cities, smart homes, manufacturing, and transport [74].

Digital twin technology provides a virtual representation of a physical product consisting of information from the product's origin to its life cycle management. The general applicability of the digital twin lies in physical entities like agricultural supply chains, automotive wiring harnesses, smart cars, and farms, and virtual entities like health monitoring and scheduling [75]. The animation concepts play a vital role in mirroring the design concepts and visualizing them during the conceptual designing stage of the digital twin [76]. For instance, while designing the speed of the machines, synchronization can be achieved by controlling the rate of animation frames [77].

Another major industrial revolution is the cyber-physical system (CPS), composed of highly integrated computation, communication, control, and physical elements. The CPS research is emerging in education [78], agriculture [79], and manufacturing. For instance, in the manufacturing sector, the CPS may bridge the gap between design and manufacturing [80]. It extends the manufacturing process with a communication interface that mimics the worker assistance system. Furthermore, the animation is used to assist the operation flow of instruments in the worker assistance system [81]. However, the CPS development is still in the embryonic stage as it faces challenges such as security, privacy, efficiency, and interoperability [82].

The interaction and fusion between the physical space and virtual space are facilitated with the advancements in the 3R technology (virtual reality (VR), augmented reality (AR), and mixed reality (MR)). The VR is a simulated environment designed in real time using computer graphics and pictures of the scene in 3D. It will immerse the viewer into the virtual environment, closing them completely away from the outside world. Meanwhile, AR is an interactive environment that is designed by increasing this fusion between the physical and virtual space. The viewers can interact with the animated data and instructional information superimposed over the real-world view through devices such as mobile phones or tablets. At the same time, the MR simulation environment is designed from the fusion of real-world and virtual space comprising the co-existence interaction of physical objects and digital objects. Two or more viewers can be networked together in a virtual environment where they can interact with computer-generated objects on the real world [83, 84]. In recent span, the AR, VR and MR applications have been widely used in health-care monitoring [84, 85], clinical applications in oral and maxillofacial surgery [86, 87], improvising nursing skills [52], and enhanced teaching strategy [27].

## 7. Conclusions

This paper highlighted a systematic review of 35 publications about animation's importance and its impact on viewer's visual attention and cognition. These publications were collected from 2015 to 2021 and are grouped into 3 categories. The risk of bias in the study design carried out in these publications was briefed. The attention-related factors such as animation motion, animated character, color, and intensity were assessed in the field of food marketing, teaching, entertainment, and advertisement. The animation motion and animated character are significant, whereas color and intensity are insignificant. The cognitive effects developed in the viewers are executive function, comprehension, spatial ability, and symbolic mediation. Meanwhile, the physical effects included confidence in their own body image and the importance of physical exercise. The limitations and recommendations associated with these 35 publications were elaborated. Also, the open challenges and issues under each category were summarized. The identified future research directions ideas may further strengthen the necessity for improving the visual quality of the animation.

The major limitation of this study is that the recently published articles were not included (i.e., publications in 2022). Several important animation research fields, such as gaming, medical, and entertainment, were not covered in this paper. Future research should include the recently published articles to enhance the quality and validate the findings in this study. In addition, the future study focuses on assessing the influence of animation motion and animated characters on the viewer's visual attention.

## Figures and Tables

**Table 1:** Comparison of existing surveys with the current review (✓: yes; x: no).

S.No	Reference	Title of research	Objective and details	Shortcomings of existing survey	Impact of animation on the viewer's visual attention and cognition	Open challenges	Future directions
1	The present study	Psychological impact and influence of animation on viewer's visual attention and cognition: a systematic literature review, open challenges, and future research directions	The impact of animation on viewers' attention and attention span was reviewed and reported either with respect to animated characters or character motion.	—	✓	✓	✓

2	Yang [12]	Research on the influence of the nature and behavior of animated characters on the audience	The influence of nature and animated characters' behavior on different audiences was analyzed. The personality classification of the animated design was discussed based on modes such as absolute justice, negative energy, and yes man mode. The study suggested that shaping character image with good temperament and behavior in animated films is necessary for guiding the children to establish a correct world outlook, outlook on life, and values.	A systematic survey protocol was not followed.	x	x	x

3	Van Rooij [13]	Carefully constructed yet curiously real: how major American animation studios generate empathy through a shared style of character design	The computer-animated characters portrayed by major American animation studios Pixar, Disney, and DreamWorks were analyzed for an overwhelming emotional response in the audience. In addition, a case study on the animation movies by these studios was carried out. The study proposed that audiences can feel equal levels of empathy for computer-animated characters and real human actors. It also suggested that the animated characters created by these studios using digital sets, virtual cameras, and perceptual cues seem to be accepted by the audience as real and authentic, and it evoked empathy.	A systematic survey protocol was not followed.	Talks about the impact on viewer's attention alone	x	x

4	Sen & Rong [14]	The influence of Japanese anime on the values of adolescent	The influence of Japanese anime on Chinese youth was elaborated. The positive and negative effects on forming correct values for adolescents were determined.	A systematic survey protocol was not followed.	x	x	x

5	Ghazali & Ghani [15]	The important of great storytelling in Malaysia animation industries	The importance of great storytelling to grab the audience's attention was exposed. Furthermore, the importance of animation story structure, such as appeal, believability, story, collaboration, and research, was elaborated.	A systematic survey protocol was not followed.	x	x	x

6	Liu & Elms [16]	Animating student engagement: the impacts of cartoon instructional videos on learning experience	The use of a series of animated videos for teaching advanced accounting at an Australian university was explored. The benefits of various demographic groups of students from these animation videos were also explored.	The design, development, and production of animated videos require more resources, and this survey did not provide a direct cost analysis.	✓	✓	✓

7	Jintapitak [16]	Use of animation characters to motivate students in a higher education class	The influence of animation on the educational field, especially for the higher education class, was explored.	A systematic survey protocol was not followed.	Talks about the impact on viewer's attention alone	x	x

8	Flynn [17]	Discovering audience motivations behind movie theater attendance	The factors that currently attract the audience to movie theaters were compared with the factors which attracted in the past. The top box office films within the past six years were selected for the study. The films were categorized into preexisting fandom, remake/sequel, superhero movies, and cinematic first.	Only a tiny portion of the top box office of all time was chosen for the study.	x	x	x

9	Zhou [18]	The narrative construction of Chinese animation from the perspective of adolescent audience	The direction of sustainable development of Chinese animation with adolescents as an object was analyzed. The characteristics of adolescence under the historical background were analyzed with respect to the phenomenon of idol worship, the dependence on networks, and the lack of knowledge of traditional culture.	A systematic survey protocol was not followed.	Talks about the impact on viewer's attention alone	x	x

10	Agarwal & Adhikari [19]	Survey of trends in 3D animation	The techniques which are in trend that are used to convert a 3D design into a 3D object on screen were analyzed. Furthermore, the techniques used to enhance state-in-art, such as texture space (continuity mapping), object space (cages), and screen space (I-Render), were elaborated.	A systematic survey protocol was not followed.	x	x	x

11	Goel & Upadhyay [20]	Effectiveness of the use of animation in advertising: a literature review	The basic concepts related to animation and its use in advertising were elaborated. Then, the animation process was discussed, including developing ideas and stories, sketching and drawing, combining it, and final editing. Also, animation styles like Walt Disney, Warner Brothers, and Japanese styles were briefed. Finally, various factors influencing the effective use of animation like attention, memory, recall and recognition, click-through rate, and attitude were discussed.	A systematic survey protocol was not followed.	Talks about the impact on viewer's attention alone	x	x

12	Zaky [21]	Once Upon a Time, We Were All Little Kids Too!!! Influence of Cartoon on Children's Behavior; Is it Just a World of Fantasy or a Nightmare???	The influence of cartoons on toddlers and children's behavior was investigated. The cartoon's beneficial effects (independent learning and proper communication) and possible harmful effects (behavior, social, and emotional development) were discussed.	A systematic survey protocol was not followed.	x	x	x

**Table 2:** Selection criteria.

S.No	Criteria	Selected criteria
1	Topic	Impact of animation on viewer's attention
2	Language	English
3	Year of Publication	2015 or later
4	Journals and conferences	Any
5	Scientific	Academic articles such as a published thesis or peer-reviewed articles

**Table 3:** Design quality analysis.

S.No	Criteria	S.No	Criteria
1.	Randomization	2.	Missing data
3.	Control	4.	Power analysis
5.	Isolation	6.	Validity measures
7.	Pre- and post-test	8.	Baseline method comparison
9.	Retention	10.	Follow up

**Table 4:** Article sources and number of articles.

S.No	Search databases	URL	No. of articles	Percentage (%)
1	Google Scholar	https://www.scholar.google.com/	199	70.82
2	Springer	https://www.springer.com/	19	6.76
3	Science Direct	https://sciencedirect.com/	18	6.41
4	Taylor & Francis	https://taylorandfrancis.com/	17	6.05
5	IEEE Xplore	https://www.ieeexplore.ieee.org/	12	4.27
6	ResearchGate	https://www.researchgate.net/	9	3.20
7	Wiley Online Library	https://onlinelibrary.wiley.com/	7	2.49
		*Total*	*281*	*100.00*

**Table 5:** Summary of animation studies.

Reference	Study description	Sample and design	Types of animation	Outcomes	Duration of animation	Findings	Case control
[25]	The audience's interaction with digital characters and the emotional impact of such films were examined. Undergraduate students of film, media, communication, and psychology courses from the Southwestern United States have participated in the study.	*n* = 144 (65 male and 79 female) Mean age: 20.6 years Design not reported	Computer-generated imagery (CGI)	Psychological outcome	60-100 min	CGI is very effective in creating a scene with characters featuring an actor either alive or passed away. In addition, it enhanced their parasocial interaction and relatability with such characters.	Real condition (*n* = 49): real human characters played by real actors CGI condition (*n* = 44): all-CG characters Hybrid condition (*n* = 51): a hybrid of characters with real humans and CG

[26]	A qualitative analysis was performed to learn the visual impacts of stop motion animated films towards the audience.	*n* = 9 (gender not mentioned) Age: 21-23 years Qualitative analysis	Stop motion	Psychological outcome	NR	The participant unknowingly noticed the techniques behind the making of stop motion film. Some of them even observed the sense of space in them.	NA

[27]	The comprehension reading of children was investigated by making them read an augmented reality (AR) storybook and was compared with those reading its printed version. The participants were selected from five children's libraries in Tehran.	*n* = 34 (20 girls and 14 boys) Age: 7-9 years Quasi-experimental methodology	2D animation AR	Cognitive outcome	NR	The AR storybooks with more interactive 3D images with added value can be used as a tool to support children's literacy learning.	Experimental group (*n* = 18; 11 girls and 7 boys): read AR storybooks Control group (*n* = 16; 9 girls and 7 boys): read the printed version of the same book

[28]	The influence of watching an animated show with fantastical events on the Chinese preschooler's executive function (EF) was investigated. The preschoolers from urban public schools in central China participated in the study.	Experiment 1 *n* = 90 (41 girls and 49 boys) Mean age: 60.37 months Latin square design Experiment 2 *n* = 20 (9 girls and 11 boys) Mean age: 63.94 months Eye-tracking technology Experiment 3 *n* = 20 (9 girls and 11 boys) Mean age: 63.94 months Strengths and difficulties questionnaire	3D animation	Cognitive outcome	Experiments 1 and 2 19 min 25 s (low fantasy) 18 min 37 s (high fantasy) Experiment 3 6 min 50 s (low fantasy and high fantasy)	The animated videos with high fantastical events have a negative impact on the preschooler's executive function.	Experiment 1 (30 participants each): high fantasy, low fantasy, no viewing Experiment 2 (10 participants each): high fantasy and low fantasy No control group Experiment 3 (10 participants each): high fantasy and low fantasy No control group

[29]	The study investigated the effectiveness of employing animated films as a measure of microintervention in a possible way to improve children's body image. Participants were selected from eastern, central, and western regions of six major US cities through a commercial research agency.	*n* = 1,329 (41% girls and 59% boys) Age: 7-14 years Randomized controlled trial	3D	Physical outcome	60 s	Microintervention effects played an essential role in creating awareness in adolescents who concern more about their body image.	Appearance teasing and bulling animation showing positive appearance self-talk (*n* = 442), media and celebrities animation showing unrealistic social media images (*n* = 441), and active control showing no body image (*n* = 446)

[30]	An interactive computer-animated agent (prototype) was developed to provide information on breast density to the women. The effectiveness of the prototype was assessed for its approval. Mammography-eligible English-speaking women were selected for the study.	*n* = 44 (all female) Age: 40-74 Cross-sectional study	Computer animation	Psychological outcome	3 min	The computer-animated agent delivers satisfied informational needs of women regarding breast density. It can be more beneficial if it is designed to deliver the psychological needs of the women undergoing the diagnosis.	NA

[31]	The changes in the student attitudes toward physical activity were evaluated by implementing Brain Breaks®videos for four months. The participants are primary grade students (3rd, 4th, and 5th) from 16 schools like Croatia, Lithuania, Macedonia, Poland, Romania, Serbia, South Africa, and Turkey.	*n* = 3,036 (1,496 males and 1,540 females) Quasi-experimental design	NR	Physical outcome	3-5 min 2 times per day 5 days each week	The student's attitude towards physical health and self-efficacy in doing exercises improved.	Experimental group (1914 participants) participated in group activity exercises and Brain Breaks®videos, and the control group (1122 participants) standard teaching materials

[32]	The effect of children's reactions towards unhealthy versus healthy products was investigated. The children's visual attention, such as dwell time and pupil dilation, was measured. The children from primary school in Austria participated in the study.	*n* = 68 (34 boys and 34 girls) Age: 6-11 years Fruit condition *n* = 34 Candy condition *n* = 34 Eye-tracking technology	2D animation cartoon style	Psychological outcome	6 min	The attractive and cartoonistic media presentation did not automatically influence the children's visual attention or emotional arousal toward unhealthy foods. However, it may prompt them when there is a restriction on taking healthy foods imposed by the parents at home.	NA

[33]	The adoption of 3D animation as a teaching tool for illustrating surgical skills in medical education was investigated. The 3D animation was hosted on Moodle platform. Third- and fifth-year medical students from the University of Botswana have participated in the study.	*n* = 90 (gender not mentioned) Age: not mentioned Randomized comparative study	Motion graphics in the form of 3D animation	Cognitive outcome	8 mins	The students preferred the adoption of 3D animation and the traditional teaching method.	Group A (traditional teaching group) is the control group, and group B (3D animation teaching group) is the experimental group

[34]	The reward learning mechanism in an infant's visual behavior was investigated. Participants are the infants recruited from the community via ads and birth records.	*n* = 51 (23 females and 28 males) Mean age: 7 months Eye-tracking technology	Animation using Adobe flash	Cognitive outcome	8 s ∗ six videos 2 times per session	Reward learning of the infant is significantly observed in the infant's mother. It played an essential role in enhancing the early cognitive development of infants.	NA

[35]	The influence of animated storybooks (motion and sound) on preschoolers' visual attention and Mandarin language learning was investigated. Children from 21 kindergartens of PAP community foundation, Singapore, were selected for the study.	*n* = 102 (49 boys and 53 girls) Age: 4-5 years Eye-tracking technology	ebook motion animation	Cognitive outcome	245 sec	The animated ebooks featuring sound and motion facilitate the children's attention, enhancing story comprehension and word learning.	Animation ebook reading group, static ebook with sound, static ebook with no motion and no sound and no reading exposure control group

[36]	The observation of hand action in visualizing the animation and static graphics was investigated. Also, the respective influence on learning the hand manipulative tasks was explored. The psychology students from Erasmus University Rotterdam have participated in the study.	*n* = 100 (20 males and 80 females) Mean age: 20.26 years	Animations were recorded in video clips	Physical outcome	NR	In learning hand manipulative tasks (i.e., knot tying), the animation delivers better knowledge than static graphics. The motor task performance was not hindered by hand appearance.	Animation with hands (*n* = 24), animation without hands (*n* = 25), statics with hands (*n* = 25), and statics without hands (*n* = 26)

[37]	The possible application of traditional animation principles to photorealistic animated animal characters was analyzed. The influence of varying degrees of exaggeration on perceived believability was investigated.	*n* = 82 (gender not mentioned) Age: 18 years Randomized block design	3D	Psychological outcome	18 sec	The animated characters should be presented realistically to achieve a higher level of the audience's perception of believability and appeal	No exaggeration, low exaggeration, high exaggeration

[38]	The influence of learning Japanese using an interactive manga-based ebook on the university students' visual attention and learning performance was investigated. The university students from the applied foreign languages department at the university in Taiwan have participated in the study.	*n* = 60 (gender not mentioned) Age: NR Eye-tracking technology	2D graphics (annotation animation)	Physical outcome	NR	Overall, students spent more time reading text and annotation than graphic information.	High prior knowledge (PK) group (*n* = 30) and low prior knowledge group (*n* = 30)

[39]	The effectiveness of using animation and static pictures to support the learning of genetics was assessed and compared. Seventh-grade students from a public junior high school participated in the study.	*n* = 181 (gender not mentioned) Age: NR	2D animation	Cognitive outcome	40 min	The animation approach is an easy and effective way to help students learn invisible infinitesimal phenomena as it lowers the perceived extraneous cognitive load.	Static pictures group (*n* = 89) and animation group (*n* = 92)

[40]	The influence of attention cueing on the learner's prior knowledge of cognitive load was investigated. Participants were students from a technology university in southeastern China.	*n* = 55 (7 male and 48 female) Mean age: 19.85 years Quasi-experimental design	Adobe flash-based animation	Physical outcome	255 sec	The interactive features of the animation help to explore knowledge	Animation only group (*n* = 27) Animation plus cueing group (*n* = 28)

[41]	The influence of characteristics of eSports virtual ads on viewers' attention was examined. It also determined how the dynamic nature of gameplay can influence those, as mentioned earlier.	*n* = 114 (40 female and 74 male) Mean age: 22.83 Eye-tracking technology	Ad animation	Psychological outcome	5 min	The advertising practitioners should take care of virtual ad designs for eSports and their placing timing.	Static condition (*n* = 58) and animated conditions (*n* = 56).

[42]	Infants' responsiveness to social attention was observed by providing training on essential attentional functions. The participants were recruited from a population-based database from the Tampere area in Finland.	*n* = 70 (37 females and 33 males) Age: 9 months Eye-tracking technology	NR	Cognitive outcome	NR	Basic training on visual attention plays an essential role in the early development of socio-cognitive skills in infants. It helps to increase the infant's responsiveness to social-communicative cues.	Training group (*n* = 35; 19 females, 16 males): four gaze-interactive games in terms of attention switching, visual search, sustained attention, and interference control and control group (*n* = 35; 18 females, 17 males), watching noncontingent, child-appropriate animations, and television clips

[43]	The virtual audiovisual environment with animated characters was employed to study hearing aid benefits. The influence of visual cues on the head and eye movements during listening talks in such an environment was investigated. Participants were young normal hearing students from the Oldenburg University.	*n* = 14 (7 males and 7 females) Age: 19-35 years	3D animation	Physical outcome	NR	The realistic animation condition was more comfortable investigating the effects of hearing aid signal processing on motion behavior. Also, it helps to identify the limitations of the technology developed.	NA

[44]	The experiences of pleasantness in viewer's emotions which stimulates the perception of pleasure portrayed in Malaysian animated cartoon characters were investigated.	*n* = 143 (78 male and 65 female) Age: 17-27 years Questionnaire's survey	3D animation	Psychological outcome	NR	During the early stage of animation production, the relationship between a character's theme and the character's appearance plays a significant role	NA

[45]	An attempt was made to increase the knowledge of glaucoma patients through animation. The factors influencing the knowledge level of such patients were determined. The patients who were diagnosed with glaucoma for six months at the King Khaled Eye Specialist Hospital were included in the study.	*n* = 196 (108 males and 88 females) Mean age: 55.7 ± 15.5 years Self-identification	Motion graphics	Psychological outcome	3 min	The animated video was influential in spreading the knowledge of glaucoma among its patients. The video should contain more information regarding the importance of long-term follow-ups with an ophthalmologist.	NA

[46]	The influence of sponsorship signage's animation intensity on the sports viewer's attention was investigated. The arousal of the viewer's confusion due to increased levels of animation intensity was explored. Participants were undergraduate and graduate sports students from the German Sport University Cologne.	*n* = 52 (40.4% female and 59.6% male) Mean age: 24.98 years Eye-tracking technology	Flash	Psychological outcome	3 min 19 sec	The visual animation has drawn sports viewers' attention to sponsorship signage even in an attractive sports environment. The animation intensity played a significant role in attracting the sports viewer's attention.	NA

[47]	The infants' recognizing ability to facial expression changes were investigated. The participants were infants recruited through newspaper ads.	*n* = 25 (15 boys and 10 girls) Mean age: 226 days Principles of the Helsinki Declaration	NR	Physical outcome	1,800 ms	The infants' ability to recognize expression was disturbed by dynamic change of facial identity using dynamic morphing animation.	Pre- and post-familiarization tests were conducted under two conditions: expression test condition and ID test condition

[48]	The audience's response to the uncanny valley, while observing different types of 3D animated characters were assessed. Also, the audience's response towards the animation films rendered in different styles was evaluated.	*n* = 50 (gender not mentioned) Age: 18-23 years Volunteer sampling	3D Computer-generated and motion capture	Psychological outcome	3 min	The participants could not feel the warmth of the real human character.	NA

[49]	The significant effect on learning idioms through English animated movies was investigated. Iranian intermediate EFL learners were selected randomly from the English language institutes in Sari for the study.	*n* = 40 (all female) Age: 14-18 years Oxford Placement Test	Animated movies	Cognitive outcome	NR	Animated movies can motivate the learners to understand the idioms in a much better way.	Experimental group (*n* = 20) provided idioms using English animated movies, and the control group (*n* = 20) exposed to those during the instruction session

[50]	The usefulness of Sasang typology in providing a theoretical backbone to the animation industry was investigated. The biopsychological features of seven animated characters in Pororo the Little Penguin were analyzed. The analysis was validated with the standardized measures of Sasang typology. The graduate school students from Pusan National University were selected for the analysis.	*n* = 41 (17 males and 24 females) Mean age: 30.64 ± 9.08 (male) and 28.62 ± 4.08 (female) Body mass index (BMI) Sasang Personality Questionnaire (SPQ)	3D animation cartoon	Physical outcome	15 min	The SPQ and BMI help analyze the biopsychosocial features as well as patients. It remained a valuable tool to educate health-care professionals and the general public regarding Korean medicine.	NA

[51]	The impact of licensed cartoon characters on children's attention to healthy food/beverages packages and their preferences was investigated. Participants were children aged 6-9 years selected via online and in-person techniques in 2012 and 2013.	*n* = 149 (gender not mentioned) Age: 6-9 years Eye-tracking technology	2D	Psychological outcome	60 trials	Healthy food and beverage packages with featured cartoon characters may enhance the children's attention and product choice.	NA

[52]	The pharmacology interleaved learning virtual reality (PILL-VR) simulation was developed to learn medication administration procedures. Its effectiveness was evaluated by employing it in nursing education.	*n* = 129 (97 female and 32 male) Mean age: 23 ± 3 years Quasi-experimental design	3D virtual reality	Psychological outcome	45 min	The VR simulations provide an affordable and flexible environment to practice medical administration. The learning practice may be made more accessible by improving students' sense of control.	Experimental group (*n* = 82; 67 female and 21 male) learned via PILL-VR environment for 3 h Comparison group (*n* = 47; 36 female and 11 male) learned via normative lecture with PowerPoint presentation for 3-4 h

[53]	The 12th grade female students from Ad Dakhiliyah Governorate in the Sultanate of Oman participated in the study. The corresponding students' spatial ability and scientific reasoning skills were observed.	*n* = 60 (all female) Mean age: NR Quasi-experimental design	2D and 3D	Cognitive outcome	8 weeks	Visualizing the chemistry concepts in 2D and 3D enhanced the spatial ability and reasoning skills of the participants	Experimental group (*n* = 32) and control group (*n* = 28).

[54]	The influence of animated film culture on the child's involvement in extended meditation in animated films is derived. Participants were preschoolers from Moscow kindergarten.	*n* = 50 (gender not mentioned) Age: 6-7 years	Animated films	Cognitive outcome	NR	Animated films enhance the child's capacity for symbolic mediation and their level of arbitrary behavior.	NA

[55]	The five-year-old children's visual perception development using 3D animated movies and interactive applications was investigated. The children from two kindergartens in Turkey participated in the study.	*n* = 38 (22 girls and 16 boys) Age: 5 years Test of visual perceptual skills-3	3D	Physical outcome	10 min Every 15 days for 16 weeks	The 3D animated movies, as well as interactive applications such as worksheets and touchscreen, can enrich the visual perceptual development of infants	Test group 1 (*n* = 12; 7 girls and 5 boys) trained with 3D animated movies and interactive application with computer Test group II (*n* = 12; 7 girls and 5 boys) trained with 3D animated movies and worksheets of the interactive application Control group (*n* = 14; 8 girls and 6 boys) trained with preschool program

[56]	The audience's perception of animated cartoons telecasted on television for political communication was investigated. The frequency of exposure to such cartoons among males and females and the audience with primary, secondary, and tertiary levels of education was determined.	*n* = 357 (182 male and 175 female) Multistage sampling	TV animated cartoons	Psychological outcome	NA	Females are more exposed to animated TV cartoons. Furthermore, the perception depends on the level of education. The cartoons should be in such a way to motivate voting behavior.	NA

[57]	The influence of animated storybooks (motion) on children's visual attention and story comprehension was investigated. Participants were children from three public schools in the Netherlands.	*n* = 39 (22 boys and 17 girls) Mean age: 61.26 months Eye-tracking technology	2D	Psychological outcome	120 s	The motion in animated illustrations caused the children to focus longer and steadily, which enhanced their capability of retelling the stories.	Storybook with animated illustrations, a storybook with static illustrations, and a control condition (only post-testing and no reading).

[58]	The influence of pedagogical agents with cueing on the students' learning ability was investigated. Seventh-grade students from a large junior high school in Taipei, Taiwan, have participated in the study.	*n* = 133 (67 boys and 66 girls) Age: 12 years	3D animation	Cognitive outcome	40 min	Implementing a pedagogical agent with cueing may help reduce the complexity of animation. It may support the learners to have a clear-cut view of the complex concepts of the biology domain.	Experimental group (*n* = 64): animation with a pedagogical agent and control group (*n* = 69), animation without a pedagogical agent

[59]	The impact of color and animation types on the sports viewer's attention to sponsorship signage was investigated. The arousal of confusion among sports viewers due to the animated sponsor signage was analyzed. Participants were assigned to four highlighted video clips (soccer, handball, biathlon, and formula one) according to their treatment conditions.	*n* = 176 (56.3% male and 43.7% female) Mean age: 24.4 ± 5 years Eye-tracking technology	Flash	Psychological outcome	15-20 min	Attention measures are more important than exposure quantities while designing sports sponsorship signage boards	Animation treatment (blinking, running, twisting, and spotlight) and color treatment (four chromatic primary hues such as red, green, blue, and yellow).

**Table 6:** Risk of bias within the studies.

Article	Randomization	Control	Isolation	Pre- and post-test	Retention	Missing data	Power analysis	Validity measure	Baseline method comparison	Follow up	Score	Reference
Sheldon et al.	Yes	No	Yes	No	No	No	No	Yes	No	No	3	[25]
Arora	Yes	No	No	No	Yes	No	No	No	No	No	2	[26]
Danaei et al.	Yes	Yes	Yes	No	Yes	No	No	No	No	No	4	[27]
Li et al.	Yes	Yes	Yes	No	Yes	No	No	No	Yes	No	5	[28]
Matheson et al.	Yes	Yes	Yes	Yes	Yes	Yes	Yes	Yes	Yes	Yes	10	[29]
Gunn et al.	No	No	Yes	Yes	Yes	Yes	No	No	Yes	No	5	[30]
Mok et al.	Yes	Yes	Yes	Yes	No	No	Yes	Yes	No	No	6	[31]
Binder et al.	Yes	No	Yes	No	Yes	No	Yes	Yes	Yes	No	6	[32]
Bedada et al.	Yes	Yes	Yes	Yes	Yes	No	No	No	No	No	5	[33]
Tummeltshammer et al.	Yes	No	Yes	Yes	Yes	Yes	No	No	Yes	No	6	[34]
Sun et al.	Yes	Yes	Yes	Yes	Yes	No	No	No	No	No	5	[35]
de Koning et al.	Yes	No	Yes	No	Yes	No	Yes	No	No	No	4	[36]
Hammer	Yes	Yes	No	No	Yes	No	No	No	No	No	3	[37]
Wang et al.	No	No	Yes	Yes	Yes	No	No	No	No	No	3	[38]
Yang et al.	Yes	No	Yes	No	No	No	No	No	No	No	2	[39]
Yang	Yes	Yes	Yes	No	No	No	No	No	No	No	3	[40]
Seo et al.	Yes	No	Yes	No	Yes	No	Yes	No	No	No	4	[41]
Forssman & Wass	Yes	Yes	Yes	Yes	Yes	No	Yes	No	No	Yes	7	[42]
Hendrikse et al.	Yes	No	Yes	No	Yes	Yes	No	Yes	No	No	5	[43]
Arshad et al.	No	No	No	No	Yes	No	Yes	No	No	No	2	[44]
Al Owaifeer et al.	No	No	No	Yes	Yes	No	No	Yes	Yes	Yes	5	[45]
Otto & Rumpf	Yes	No	Yes	No	Yes	Yes	Yes	No	No	No	5	[46]
Ichikawa et al.	Yes	No	Yes	Yes	Yes	No	Yes	No	No	No	5	[47]
Bouwer & Human	Yes	No	Yes	No	Yes	No	No	No	No	No	3	[48]
Sanaeifar	Yes	Yes	Yes	Yes	No	No	Yes	No	No	No	5	[49]
Yoon et al.	No	No	No	No	No	No	No	Yes	No	No	1	[50]
Ogle et al.	No	No	No	No	Yes	No	Yes	No	No	No	2	[51]
Dubovi et a.l	Yes	Yes	Yes	Yes	Yes	No	No	Yes	Yes	No	7	[52]
Al-Balushi et al.	Yes	Yes	Yes	Yes	No	No	No	No	No	No	4	[53]
Martynenko	Yes	No	Yes	No	No	No	No	No	No	No	2	[54]
Yucelyigit & Aral	Yes	Yes	Yes	Yes	Yes	No	No	Yes	No	No	6	[55]
Okoro & Onakpa	Yes	No	No	Yes	Yes	No	No	Yes	No	No	4	[56]
Takacs & Bus	No	Yes	Yes	No	Yes	No	Yes	No	No	No	4	[57]
Yung & Paas	Yes	Yes	Yes	Yes	Yes	No	No	No	No	No	5	[58]
Breuer & Rumpf	Yes	No	Yes	No	Yes	No	No	No	No	No	3	[59]

## Data Availability

The original contributions generated for this study are included in the article; further inquiries can be directed to the corresponding author.
